# Racial and Ethnic Disparities in Perceived Health Status Among Patients With Cardiovascular Disease

**DOI:** 10.5888/pcd21.240264

**Published:** 2024-11-14

**Authors:** Marjan Zakeri, Lincy S. Lal, Susan M. Abughosh, Shubhada Sansgiry, E. James Essien, Sujit S. Sansgiry

**Affiliations:** 1 Pharmaceutical Health Outcomes and Policy Department, College of Pharmacy, University of Houston, Texas; 2 School of Public Health, University of Texas Health Science Center at Houston; 3 Health System Research and Development Center for Innovations in Quality, Effectiveness, and Safety, Michael E. DeBakey VA Medical Center, Houston, Texas; 4 VA South Central Mental Illness Research, Education and Clinical Center, Michael E. DeBakey VA Medical Center Houston, Texas; 5 Department of Medicine, Section of Health Services Research, Baylor College of Medicine, Houston, Texas

## Abstract

**Introduction:**

Understanding health outcomes among people with cardiovascular disease (CVD) is crucial for improving treatment strategies and patient quality of life. This study investigated racial and ethnic disparities in perceived health status among non-Hispanic Black, Hispanic, and non-Hispanic White adults with CVD.

**Methods:**

The study had a retrospective cross-sectional design and used data from the Medical Expenditure Panel Survey spanning 8 calendar years (2014–2021). The study population consisted of adults diagnosed with various CVDs. We used ordinal logistic regression models adjusted for demographic and socioeconomic characteristics, CVD severity, comorbidities, and health care expenditures to assess racial and ethnic differences in perceived health status.

**Results:**

Among the 11,715 (weighted frequency, 15,431,283) adults with CVD, we observed significant differences in perceived health status across racial and ethnic cohorts. The unadjusted analysis showed that non-Hispanic Black adults had significantly higher odds than non-Hispanic White adults of perceiving their health as poorer (odds ratio [OR]= 1.89; 95% CI, 1.74–2.07; *P* < .001), with a similar observation among Hispanic adults (OR = 2.05; 95% CI, 1.85–2.26; *P* < .001). Although female sex, higher education, and better income had protective effects on perceived health status independent of race, we found significant racial and ethnic differences in the effect of older age, physical and cognitive limitations, and health insurance status on perceived health status.

**Conclusion:**

This study revealed substantial racial disparities in perceived health status among adults with CVD, with notable differences in the effects of predictive factors. Addressing these disparities requires targeted interventions to improve health care access and enhance socioeconomic conditions tailored to the needs and experiences of racial and ethnic populations.

**Summary**

**What is already known on this topic?**

Racial and ethnic disparities in perceived health status exist among people with cardiovascular disease, with Black and Hispanic people often reporting poorer health than White people.

**What is added by this report?**

Although sex, education, and income level have similar effects on perceived health status independent of race, significant racial and ethnic differences exist in the effects of older age, physical and cognitive limitations, and insurance status on perceived health status.

**What are the implications for public health practice?**

Targeted interventions addressing socioeconomic factors, enhancing education, and improving access to specialized health care can mitigate disparities in perceived health status and promote better health outcomes in racial and ethnic minority populations with cardiovascular disease.

## Introduction

Understanding health outcomes among people with cardiovascular disease (CVD) is crucial for improving treatment strategies and patient quality of life. CVD is a leading cause of illness and death globally, necessitating comprehensive research into factors influencing health outcomes. Despite extensive studies on CVD, a gap remains in the literature concerning racial and ethnic differences in CVD health outcomes, among which perceived health status is of great importance (1–3).

A recent analysis of 3 randomized clinical trials found no significant differences in the efficacy of sodium-glucose cotransporter-2 inhibitors (a class of antihyperglycemic drugs that help lower blood glucose levels in adults with type 2 diabetes) between Black and White patients with heart failure. However, after adjusting for age, sex, and trial, Black patients had significantly worse baseline scores than White patients on the Kansas City Cardiomyopathy Questionnaire in several domains (4). Another clinical trial using the University of Washington Quality of Life Questionnaire found that, after adjusting for confounders, Black race was an independent predictor of poorer quality of life among cancer patients (5). A cross-sectional study of 806 stroke survivors from the 2011 National Health and Aging Trends Study found that Black study participants had lower physical capacity and more activity limitations than White study participants (mean 5.1 vs 6.9, *P* < .01), even after adjusting for sociodemographic factors and comorbidities (6). Moreover, poor health outcomes were also observed in EuroQol Group measures for Hispanic stroke survivors compared with their non-Hispanic counterparts, as reported in a study using 2000 and 2002 Medical Expenditure Panel Survey data (7). These findings highlight a gap in the identification of racial and ethnic disparities in perceived quality of life.

This study had a dual objective: first, to assess the extent of racial and ethnic disparities in perceived health status among adults with CVD, and second, to examine the factors influencing perceived health status in racial and ethnic cohorts.

## Methods

The study used a retrospective cross-sectional design and focused on a sample of adults aged 18 years or older diagnosed with CVD. We collected data from the Medical Expenditure Panel Survey (MEPS) (8). MEPS collects comprehensive data on health care expenditures, usage trends, and health insurance coverage from US households, individuals, health care providers, and employers. MEPS has 4 main components: the Household Component, the Medical Provider Component, the Insurance Component, and the Nursing Home Component. The Household Component is the survey’s primary component. MEPS uses a stratified, multistage sampling method with disproportionate selection, enabling researchers to produce national estimates (9).

### Inclusion and exclusion criteria

**Figure 1:**
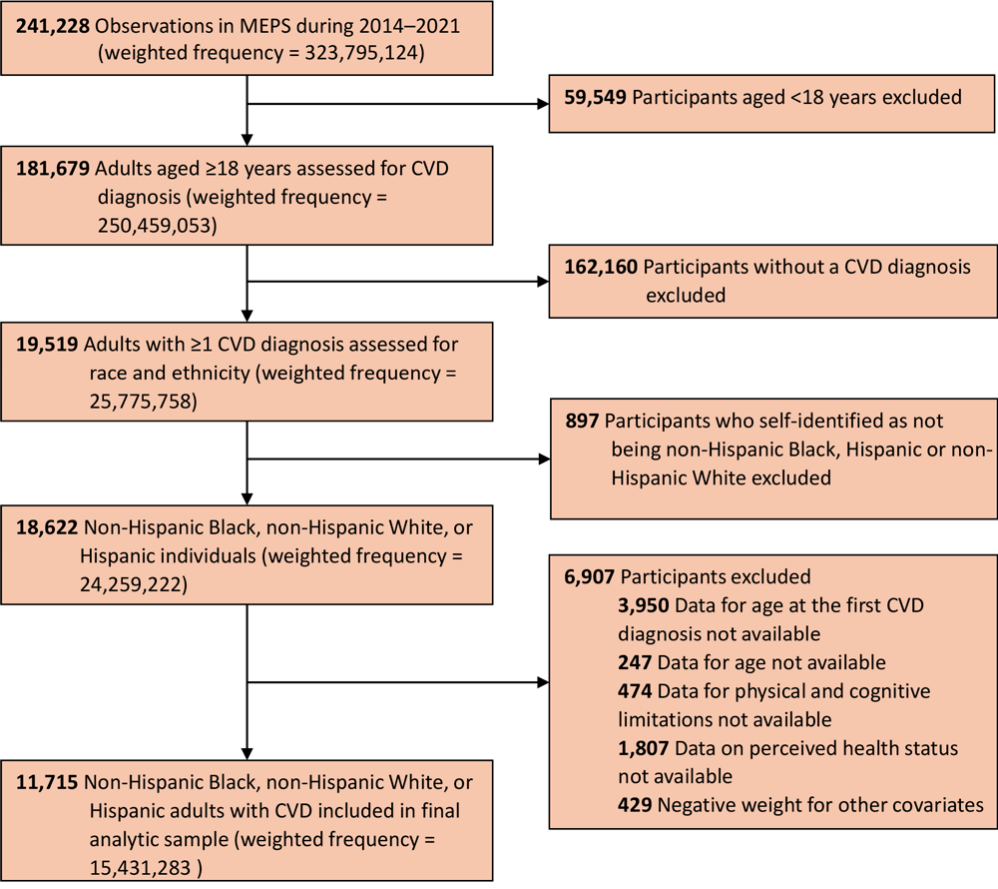
Flowchart showing the process of determining the number of adults included in a study on racial and ethnic differences in perceived health status among adults with CVD. Data are from MEPS, 2014–2021. Abbreviations: CVD, cardiovascular disease; MEPS, Medical Expenditure Panel Survey.

The study population included Hispanic, non-Hispanic Black (hereinafter referred to as Black), and non-Hispanic White (hereinafter referred to as White) adults diagnosed with CVD (Figure 1). The analysis excluded other racial and ethnic groups due to their small sample sizes. The included CVDs were ischemic heart disease, cardiomyopathy, cardiac arrest, peripheral artery disease, heart failure, cardiac arrhythmia, heart valve issues, and stroke (10,11) Codes from the *International Classification of Diseases, Ninth Revision, Clinical Modification* (ICD-9-CM [12]) and *International Classification of Diseases, Tenth Revision, Clinical Modification* (ICD-10-CM [13]) (Appendix Table 1) were used to identify CVD conditions. We excluded adults with missing data for study covariates or outcomes.

### Measures

**Figure 2:**
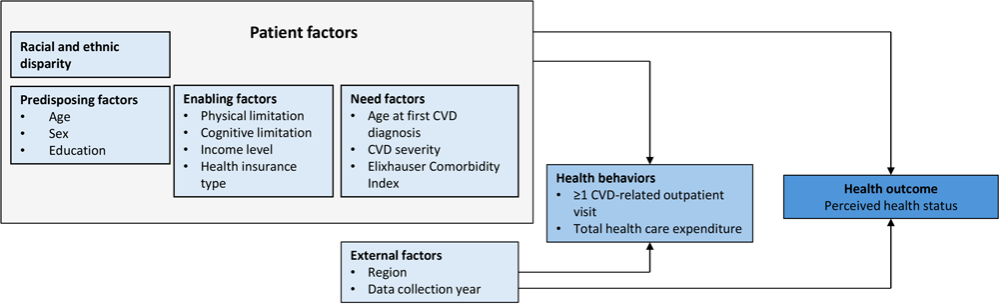
Proposed conceptual model based on Andersen’s Behavioral Model of Health Services Use (14). Abbreviation: CVD, cardiovascular disease.

We used Andersen’s Behavioral Model of Health Services Use to select the following key elements: predisposing factors, enabling factors, need factors, external factors, and health behaviors, all of which influence health outcomes (Figure 2) (14). The Andersen model primarily focuses on understanding health care use rather than the quality of care or health outcomes. Therefore, although our model adjusted for total health care expenditures, it did not fully capture aspects related to quality of care received. This distinction is crucial because the model may not comprehensively reflect how health care experiences affect perceived health-related quality of life.

The outcome variable in this study was perceived health status (MEPS variable name: RTHLTH53). This question asked the respondent to rate their health status according to the following categories: excellent, very good, good, fair, and poor.

We included data on the following demographic and socioeconomic characteristics: race and ethnicity (Black, Hispanic, White), sex (male, female), age (18–44, 45–64, 65–69, ≥70 y), educational attainment, annual family income, health insurance status, and physical and cognitive limitations. External factors were geographic region (Northeast, Midwest, South, and West) and data collection year (2014–2021). Educational attainment was categorized into high school diploma or less, 1 to 3 years of college, and 4 or more years of college.

MEPS uses income categories defined by poverty statistics from the Current Population Survey, sponsored jointly by the US Census Bureau and the US Bureau of Labor Statistics. Annual family income was classified as a percentage of the poverty threshold for that year, grouping survey participants into 5 categories: poor (<100%), near poor (100% to <125%), low income (125% to <200%), middle income (200% to <400%), and high income (≥400%). For this study, we combined the poor and near-poor categories into a single poor/near-poor category because of small sample sizes.

Physical limitations were identified through a MEPS family-level question: “Does anyone in the family have difficulties walking, climbing stairs, grasping objects, reaching overhead, lifting, bending or stooping, or standing for long periods of time?” If the answer was yes, the next question identified respondents with physical limitations.

Cognitive limitations were assessed by using a 3-part MEPS family-level question: “Does any adult in the family 1) experience confusion or memory loss, 2) have problems making decisions, or 3) require supervision for their safety?” If the answer was yes to any of these questions, the next question identified respondents with cognitive limitations.

Health insurance status was categorized into 3 groups. The “any private” category included adults with private insurance from sources such as employer or union plans, private insurance obtained through federally facilitated or state-based exchanges or marketplaces, and TRICARE/CHAMPVA. The “only public” category comprises adults covered solely by public programs such as Medicare (parts A and B), Medicaid, other public hospital/physician programs, or the Veterans Administration. Finally, adults without any health insurance were categorized as uninsured.

The study integrated 3 critical measures related to need: age at CVD diagnosis, CVD severity score, and the Elixhauser Comorbidity Index (15,16). Age at CVD diagnosis was defined as the earliest age at which any CVD was clinically identified in the adult’s medical history, as recorded in the Medical Provider Component dataset. The CVD severity score, adapted from a previously established scoring system (15), was refined to accurately quantify the severity of CVD conditions within the limitations of our dataset (Appendix Table 2). Because of the 3-digit ICD coding in MEPS, we made refinements to avoid potential duplications. For example, we removed code 427 (cardiac dysrhythmias) and code 424 (valve disorders) when necessary to prevent double counting. Additionally, because use of a pacemaker or defibrillator is classified as a procedure rather than a diagnosis, and MEPS does not include this information, we excluded it from the CVD severity score. Lastly, we created a modified version of the Elixhauser Comorbidity Index (16). We made this modification because MEPS data are limited to 3-digit ICD codes, which made it impossible to differentiate between some conditions. Specifically, we could not distinguish between uncomplicated and complicated diabetes or between anemia due to blood loss and anemia due to iron deficiency. To avoid double counting and ensure accurate analysis, we modified the Elixhauser Comorbidity Index by combining these indistinguishable conditions, resulting in a total of 29 conditions included in our study. We used the modified Elixhauser Comorbidity Index and the CVD severity score as separate variables. This approach allowed us to independently evaluate the effects of general comorbidities and CVD severity on patient outcomes.

Lastly, we used 2 measures to adjust for use of CVD-related health care resources and overall health care expenditure. The first measure, termed “CVD visit,” was a binary variable indicating whether at least 1 CVD-related outpatient visit occurred during the study period. The second measure focused on total health care expenditure for each MEPS participant with CVD during the study period. To maintain temporal consistency, all medical expenditure estimates from 2014 to 2020 were adjusted to the 2021 dollar value by using the medical care component of the Consumer Price Index, as provided by the US Bureau of Labor Statistics (17).

### Statistical analysis

We used data spanning 8 years (2014–2021) from the Household Component and Medical Provider Component datasets. After compiling 8 years of data, we used the proper variance structure for making estimates from MEPS data that were pooled over multiple years (18). In this regard, STRA9621 (stratum) and PSU9621 (primary sampling unit) variables from the Household Component-036 file were merged with the primary data set to facilitate the generation of annual estimates (18).

We performed a sample size calculation to ensure sufficient power to detect even small effects in the presence of multiple predictors. We used G*Power 3.1 software (19) to conduct an a priori power analysis for a logistic regression model. Given the complexity of our model, which includes numerous predictors and interaction terms, we set a conservative estimate for the proportion of variance explained by other predictors (*R^2^* = 0.90) to capture potential small effects. We aimed for a power of 0.80, with an OR of 1.3 and a significance level of .05. The analysis indicated that a total sample size of 7,208 participants would be required to achieve the desired power level. Our sample consisted of 11,715 participants (weighted frequency, 15,431,283), ensuring that the study was more than adequately powered to detect the effects of interest.

For the total health care expenditure outcome, we added $1 to all observed expenditures, followed by log transformation to normalize the data. We chose the log transformation of total expenditure over raw expenditure to address nonlinearity, capture diminishing marginal utility, standardize scale, and ultimately enhance the robustness and interpretability of our findings.

We conducted descriptive weighted analyses to compare the characteristics of adults with CVD across racial and ethnic groups. We used ordinal logistic regression to properly address the ordered outcome and assess the odds of reporting poorer perceived health status among adults diagnosed with CVD. We added interaction terms to the adjusted model to investigate further the effects of covariates both between and within racial groups. 

To mitigate the influence of the COVID-19 pandemic, we conducted sensitivity analyses after excluding data from 2020 and 2021. We used SAS version 9.4 (SAS Institute Inc) to perform all statistical analyses. Approval for the study was secured from the institutional review board at the University of Houston.

## Results

We observed racial differences for most covariates, including age, sex, educational level, physical and cognitive limitations, health insurance type, income level, and regional distribution (Table 1).

**Table 1:** Characteristics of Adults With CVD (N = 11,715), Medical Expenditure Panel Survey, 2014–2021

Characteristic	Weighted frequency (%)^a^			*P* value^b^
	Black (n = 2,113)	White (n = 8,079)	Hispanic (n = 1,523)	
**Age, y**				
18–44	133,113 (8.2)	587,210 (4.7)	114,786 (9.0)	<.001
45–64	624,360 (38.4)	3,345,713 (26.7)	499,336 (39.2)	
65–69	262,541 (16.1)	1,778,612 (14.2)	187,818 (14.7)	
≥70	607,157 (37.3)	6,818,434 (54.4)	472,202 (37.1)	
**Sex**				
Male	763,871 (46.9)	7,003,937 (55.9)	653,177 (51.3)	<.001
Female	863,301 (53.1)	5,526,032 (44.1)	620,965 (48.7)	
**Educational attainment**				
High school diploma or less	1,146,164 (70.4)	7,202,387 (57.5)	900,873 (70.7)	<.001
1–3 years of college	298,837 (18.4)	2,453,308 (19.6)	228,609 (17.9)	
≥4 years of college	182,171 (11.2)	2,874,274 (22.9)	144,661 (11.4)	
**Has a physical limitation**				
Yes	860,150 (52.9)	5,273,972 (42.1)	533,815 (41.9)	<.001
No	767,022 (47.1)	7,255,998 (57.9)	740,327 (58.1)	
**Has a cognitive limitation**				
Yes	441,789 (27.2)	1,988,757 (15.9)	261,637 (20.5)	<.001
No	1,185,383 (72.8)	10,541,212 (84.1)	1,012,505 (79.5)	
**Health insurance type**				
Any private	624,285 (38.4)	7,414,993 (59.2)	463,667 (36.4)	<.001
Only public	961,116 (59.1)	4,947,970 (39.5)	744,890 (58.5)	
Uninsured	41,771 (2.6)	167,006 (1.3)	65,585 (5.1)	
**Income level^c^**				
Poor/near poor	606,858 (37.3)	1,914,076 (15.3)	397,463 (31.2)	<.001
Low income	284,997 (17.5)	1,808,954 (14.4)	252,947 (19.8)	
Middle income	394,317 (24.2)	3,579,713 (28.6)	340,653 (26.7)	
High income	340,999 (21.0)	5,227,227 (41.7)	283,080 (22.2)	
**Region of residence**				
Northeast	225,899 (13.9)	2,392,231 (19.1)	264,488 (20.8)	<.001
Midwest	303,719 (18.7)	3,278,438 (26.2)	125,868 (9.9)	
South	974,641 (59.9)	4,722,043 (37.7)	453,702 (35.6)	
West	122,912 (7.6)	2,137,257 (17.1)	430,085 (33.8)	
**Data collection year**				
2014	225,042 (13.8)	1,836,264 (14.6)	174,219 (13.7)	.84
2015	253,264 (15.6)	1,895,165 (15.1)	174,129 (13.7)	
2016	238,829 (14.7)	1,771,500 (14.1)	183,429 (14.4)	
2017	221,589 (13.6)	1,518,672 (12.1)	184,341 (14.5)	
2018	212,663 (13.1)	1,690,294 (13.5)	182,201 (14.3)	
2019	186,191 (11.4)	1,588,455 (12.7)	149,131 (11.7)	
2020	117,802 (7.2)	944,988 (7.5)	90,960 (7.1)	
2021	171,793 (10.6)	1,284,632 (10.2)	135,731 (10.1)	
**≥1 CVD-related outpatient visit during the study period**				
No	358,281 (22.0)	2,328,585 (18.6)	263,640 (20.7)	.05
Yes	1,268,891 (78.0)	10,201,384 (81.4)	1,010,502 (79.3)	
**Perceived health status**				
Excellent	64,909 (4.0)	980,489 (7.8)	95,265 (7.5)	<.001
Very good	273,840 (16.8)	3,292,112 (26.3)	185,148 (14.5)	
Good	542,196 (33.3)	4,404,053 (35.2)	449,547 (35.3)	
Fair	534,062 (32.8)	2,702,183 (21.6)	393,260 (30.9)	
Poor	212,164 (13.0)	1,151,133 (9.2)	150,923 (11.8)	
**Other,** **mean (SE) [95% CI]**				
Age at CVD diagnosis, y	52.9 (0.6) [52.0–53.9]	57.0 (0.4) [56.4–57.6]	52.9 (0.8) [51.6–54.2]	—
CVD severity score	3.6 (0.05) [3.5–3.7]	3.4 (0.03) [3.4–3.5]	3.5 (0.1) [3.4–3.6]	—
Elixhauser Comorbidity Index	3.5 (0.1) [3.3–3.6]	3.4 (0.03) [3.3–3.4]	3.3 (0.1) [3.2–3.4]	—
Total health care expenditures, $	18,575 (777) [17,294–19,857]	17,866 (337) [17,312–18,421]	17,298 (809) [15,964–18,632]	—

Abbreviation: — , does not apply; CVD, cardiovascular disease.

a Unless otherwise indicated.

b Determined by χ^2^ test; *P* < .05 considered significant.

c Defined by poverty statistics from the Current Population Survey, sponsored jointly by the US Census Bureau and the US Bureau of Labor Statistics. Annual family income was classified as a percentage of the poverty threshold for that year, grouping survey participants into 5 categories: poor (<100%), near poor (100% to <125%), low income (125% to <200%), middle income (200% to <400%), and high income (≥400%). For this study, we combined the poor and near-poor categories into a single poor/near-poor category.

The unadjusted ordinal logistic regression indicated that Black adults had significantly higher odds, compared with White adults, of reporting poorer perceived health status (OR = 1.89; 95% CI, 1.74–2.07; *P* < .001), with a similar trend observed among Hispanic adults (OR = 2.05; 95% CI, 1.86–2.26; *P* < .001).

The adjusted ordinal regression showed significant racial differences in perceived health status (Table 2). Black adults had significantly higher odds, compared with White adults, of reporting poorer perceived health (adjusted OR [AOR] = 1.72; 95% CI, 1.03–2.88; *P* = .04). These findings suggest that Black adults with CVD perceive their health status more negatively than their White counterparts. In contrast, Hispanic adults did not have significantly higher odds, compared with White adults, of reporting poorer perceived health (AOR = 0.85; 95% CI, 0.49–1.45; *P* = .54).

**Table 2:** Adjusted Odds Ratios of Predictive Factors From Ordinal Logistic Regression Outcomes Relative to Perceived Health Status of Adults With CVD (N = 11,715), Medical Expenditure Panel Survey, 2014–2021^a^

Characteristic	Estimate (SE)	AOR (95% CI) for reporting poorer perceived health	*P* value
**Race**			
Black	0.54 (0.26)	1.72 (1.03–2.88)	.04
Hispanic	−0.17 (0.27)	0.85 (0.49–1.45)	.54
White	—	1 [Reference]	—
**Sex**			
Female	−0.17 (0.04)	0.84 (0.77–0.92)	<.001
Male	—	1 [Reference]	—
**Race × sex**			
Black female	0.12 (0.09)	1.12 (0.94–1.35)	.20
Hispanic female	0.01 (0.11)	1.01 (0.82–1.24)	.94
**Age, y**			
18–44	—	1 [Reference]	—
45–64	−0.11 (0.12)	0.89 (0.71–1.12)	.33
65–69	−0.37 (0.13)	0.69 (0.54–0.89)	.004
≥70	−0.82 (0.12)	0.44 (0.34–0.56)	<.001
**Race × age, y**			
Black			
45–64	−0.13 (0.21)	0.88 (0.58–1.31)	.52
65–69	−0.17 (0.23)	0.84 (0.54–1.31)	.44
≥70	0.15 (0.21)	1.16 (0.77–1.74)	.48
Hispanic			
45–64	0.29 (0.22)	1.33 (0.87–2.03)	.19
65–69	0.50 (0.24)	1.65 (1.02–2.65)	.04
≥70	0.67 (0.22)	1.96 (1.27–3.03)	.002
**Educational attainment**			
High school diploma or less	—	1 [Reference]	—
1–3 years of college	−0.19 (0.06)	0.82 (0.74–0.92)	<.001
≥4 years of college	−0.47 (0.05)	0.62 (0.56–0.70)	<.001
**Race × educational attainment**			
Black			
1–3 years of college	−0.03 (0.13)	0.97 (0.75–1.24)	.79
≥4 years of college	0.00 (0.16)	1.00 (0.73–1.39)	.98
Hispanic			
1–3 years of college	0.19 (0.16)	1.21 (0.89–1.65)	.22
≥4 years of college	−0.33 (0.19)	0.72 (0.49–1.05)	.08
**Has a physical limitation**			
Yes	0.97 (0.05)	2.63 (2.40–2.89)	<.001
No	—	1 [Reference]	—
**Race × Has a physical limitation**			
Black	−0.33 (0.10)	0.72 (0.59–0.88)	.001
Hispanic	−0.50 (0.12)	0.61 (0.48–0.77)	<.001
**Has a cognitive limitation**			
Yes	0.70 (0.06)	2.01 (1.79–2.26)	<.001
No	—	1 [Reference]	—
**Race × Has a cognitive limitation**			
Black	−0.10 (0.11)	0.90 (0.72–1.13)	.37
Hispanic	−0.27 (0.14)	0.76 (0.58–0.99)	.048
**Health insurance**			
Only public	0.29 (0.05)	1.34 (1.23–1.47)	<.001
Uninsured	0.99 (0.18)	2.70 (1.88–3.87)	<.001
Any private	—	1 [Reference]	—
**Race × Health insurance**			
Black			
Only public	−0.11 (0.11)	0.90 (0.73–1.11)	.32
Uninsured	−0.21 (0.32)	0.81 (0.43–1.53)	.52
Hispanic			
Only public	0.32 (0.14)	1.38 (1.06–1.80)	.02
Uninsured	0.26 (0.28)	1.29 (0.75–2.24)	.36
**Income level^b^**			
Poor/near poor	0.57 (0.07)	1.77 (1.56–2.02)	<.001
Low income	0.42 (0.07)	1.53 (1.34–1.74)	<.001
Middle income	0.22 (0.05)	1.25 (1.13–1.38)	<.001
High income	—	1 [Reference]	—
**Race × Income level^b^**			
Black			
Poor/near poor	−0.27 (0.15)	0.77 (0.57–1.03)	.08
Low income	−0.29 (0.17)	0.75 (0.54–1.03)	.08
Middle income	0.11 (0.15)	1.12 (0.84–1.50)	.45
Hispanic			
Poor/near poor	0.01 (0.18)	1.01 (0.71–1.45)	.94
Low income	0.17 (0.19)	1.18 (0.81–1.72)	.39
Middle income	0.19 (0.17)	1.21 (0.86–1.70)	.28
**Region**			
Midwest	0.01 (0.06)	1.01 (0.89–1.13)	.93
South	0.04 (0.06)	1.04 (0.93–1.17)	.47
West	0 (0.07)	1.00 (0.87–1.14)	.96
Northeast	—	1 [Reference]	—
**Race × Region**			
Black			
Midwest	−0.36 (0.16)	0.70 (0.51–0.96)	.03
South	0.04 (0.14)	1.04 (0.79–1.36)	.79
West	−0.03 (0.20)	0.97 (0.65–1.44)	.87
Hispanic			
Midwest	−0.22 (0.20)	0.80 (0.54–1.19)	.27
South	0.10 (0.15)	1.11 (0.83–1.47)	.49
West	0.04 (0.15)	1.05 (0.77–1.41)	.77
**Data collection year**			
2014	—	1 [Reference]	—
2015	−0.05 (0.07)	0.95 (0.83–1.08)	.43
2016	0.02 (0.07)	1.02 (0.89–1.17)	.77
2017	0.03 (0.07)	1.04 (0.90–1.19)	.62
2018	0 (0.07)	1.00 (0.88–1.15)	.95
2019	−0.10 (0.07)	0.90 (0.79–1.04)	.16
2020	−0.02 (0.08)	0.98 (0.84–1.16)	.83
2021	−0.04 (0.07)	0.96 (0.83–1.11)	.56
**≥1 CVD-related outpatient visit during the study period**			
No	−0.09 (0.06)	0.91 (0.81–1.02)	.10
Yes	—	1 [Reference]	—
**≥1 CVD-related outpatient visit during the study period × Race**			
Black/no visit	0.18 (0.11)	1.19 (0.96–1.48)	.10
Hispanic/no visit	−0.02 (0.12)	0.98 (0.77–1.25)	.86
**Other**			
Age at CVD diagnosis	−0.003 (0.001)	1.00 (0.99–1.00)	.053
CVD severity score	0.06 (0.02)	1.06 (1.03–1.09)	<.001
Elixhauser Comorbidity Index	0.14 (0.01)	1.15 (1.12–1.18)	<.001
Log total health care expenditure	0.25 (0.01)	1.28 (1.25–1.32)	<.001

Abbreviation: —, does not apply; CVD, cardiovascular disease.

a Adjusted ordinal logistic regression was performed to assess the odds of reporting poorer perceived health status among adults diagnosed with CVD.

b Defined by poverty statistics from the Current Population Survey, sponsored jointly by the US Census Bureau and the US Bureau of Labor Statistics. Annual family income was classified as a percentage of the poverty threshold for that year, grouping survey participants into 5 categories: poor (<100%), near poor (100% to <125%), low income (125% to <200%), middle income (200% to <400%), and high income (≥400%). For this study, we combined the poor and near-poor categories into a single poor/near-poor category.

Sex also significantly influenced perceived health status. Women had lower odds than men of reporting poorer perceived health (AOR = 0.84, 95% CI, 0.77–0.92; *P* < .001). However, the interaction between race and sex was not significant, suggesting that the sex disparity in perceived health status was consistent across racial groups.

Age was another factor affecting perceived health status. Adults aged 65 to 69 years and adults aged 70 or older had significantly lower odds of reporting poorer health compared with adults aged 18 to 44 (AOR = 0.69, 95% CI, 0.54–0.89; *P* = .004 and AOR = 0.44, 95% CI, 0.34–0.56; *P* < .001, respectively). The interaction between race and age indicated significantly higher odds of reporting poorer perceived health among Hispanic adults aged 65 to 69 and 70 years or older compared with their White counterparts (AOR = 1.65, 95% CI, 1.02–2.65; *P* = .04 and AOR = 1.96, 95% CI, 1.27–3.03; *P* = .002, respectively).

Having physical or cognitive limitations significantly increased the odds of reporting poorer perceived health status (AOR = 2.63, 95% CI, 2.40–2.89; *P* < .001 and AOR = 2.01, 95% CI, 1.79–2.26; *P* < .001, respectively). However, these effects were moderated by race. The significance of interaction terms for physical limitations (Black adults, OR = 0.72, 95% CI, 0.59–0.88; *P* = .001 and Hispanic adults, OR = 0.61, 95% CI, 0.48–0.77; *P* < .001) and cognitive limitations (Hispanic adults, OR = 0.76, 95% CI, 0.58–0.99; *P* = .048) indicated a weaker association between physical and cognitive limitations and perceived health status among Black and Hispanic adults compared with White adults.

Health insurance status was also a significant predictor of perceived health status. Those with only public insurance or who were uninsured had higher odds of reporting poorer health compared with those with private insurance (AOR = 1.34, 95% CI, 1.23–1.47; *P* < .001 and AOR = 2.70, 95% CI, 1.88–3.87; *P* < .001, respectively). The interaction terms showed significant racial differences. Hispanic adults with only public insurance had higher odds of poorer perceived health status compared with their White counterparts (AOR = 1.38, 95% CI, 1.06–1.80; *P* = .02), indicating a stronger effect among Hispanic adults compared with their White counterparts.

Income level was a significant predictor of perceived health status, with poor or near-poor adults (AOR = 1.77, 95% CI, 1.56–2.02; *P* < .001), low-income adults (AOR = 1.53, 95% CI, 1.34–1.74; *P* < .001), and middle-income adults (AOR = 1.25, 95% CI, 1.13–1.38; *P* < .001) reporting poorer perceived health status than high-income adults. The interaction terms between race and income level did not yield significant differences between different races.

Study findings were robust to sensitivity analysis with the removal of 2020 and 2021 data.

## Discussion

Our findings indicate that Black and Hispanic adults with CVD had significantly higher odds than White adults with CVD of reporting poorer perceived health. This difference remained significant for the Black cohort after adjusting for the factors in the model. Our investigation, adjusted for health conditions and comorbidities, underscores the substantial effects of demographic and socioeconomic factors on perceived health status.

The poorer perceived health status among Black adults in our study supports previous findings among patients with heart failure, where scores from the Kansas City Cardiomyopathy Questionnaire (KCCQ) were lower among Black patients (mean, 64; SD, 21) compared with White patients (mean, 67; SD, 20), even after adjusting for age, sex, educational attainment, severity of heart failure, and risk factors (20). Although the study did not delve into reasons for the poorer perceived health status, it clearly established that the KCCQ score is a strong predictor of 1-year mortality, independent of race and ethnicity (hazard ratio = 0.45; 95% CI, 0.30–0.67 for the highest vs lowest quintile of KCCQ). Our findings align with those of a recent study on patients with chronic obstructive pulmonary disease, which used data from 2016–2019 MEPS and identified a significant disparity in perceived health status between Black and White patients, with 44.3% of Black patients reporting fair or poor health compared with 35.3% of White patients (21). These results underscore the persistent disparities in health outcomes among racial and ethnic groups, emphasizing the need for targeted interventions to address these inequities and enhance patient care.

In our study, physical limitations emerged as the most significant predictor of poor perceived health status among all races, with the strongest effect among White adults. This finding aligns with previous research identifying a strong association between physical limitations and reduced quality of life (22). Physical limitations not only directly affect overall health but also may affect the perception of well-being (23). Consequently, people with physical limitations often experience a negative cycle where both their perceived physical and mental health are adversely affected (23,24). The stronger effect of physical limitations on perceived health among the White cohort might be attributed to a combination of cultural, social, psychological, and systemic factors that influence perceived health status and reporting behaviors (25).

Cognitive limitations were also a significant predictor of perceived health status across all racial groups; however, the effect was weaker in the Hispanic cohort. This finding corroborates previous research highlighting the association between cognitive limitations and quality of life (26). The cognitive limitation might affect a person’s ability to accurately interpret symptoms, communicate effectively with health care providers, and manage medications and treatment plans (27). Communication limitations often lead to increased emotional issues such as depression and anxiety, further skewing one’s perceived health status (24). Additionally, difficulties in daily functioning and social interactions can contribute to feelings of frustration, helplessness, and isolation, all of which negatively influence how individuals view their overall well-being (24). The weaker effect of cognitive limitations on perceived health among Hispanic adults in our study can be attributed to cultural perceptions, strong social support, effective coping mechanisms, reporting differences, access to resources, and prioritizing health concerns (28).

Income level also emerged as a key predictor of poorer perceived health status across all races. Poor perceived health status may result from limited access to quality health care, increased stress from financial instability, and the burden of managing health conditions with insufficient support (29,30). These economic difficulties not only affect physical health but also intensify emotional and mental stress, leading to a poorer perception of overall well-being (30).

Another significant predictor of poorer perceived health was sole reliance on public health insurance or a lack of health insurance, with a stronger effect among Hispanic adults with public insurance. This finding suggests that people who rely exclusively on public insurance might lack full access to outpatient medications or the benefits offered by Medicare Advantage plans. A review of 62 studies by the Kaiser Family Foundation highlighted differences between Medicare Advantage and traditional Medicare (Parts A and B) (31). The review found that Medicare Advantage enrollees were more likely to have a usual source of care and receive preventive services such as annual wellness visits. The stronger effect of sole reliance on public insurance among Hispanic adults in our study might be explained by language and communication challenges, health care discrimination, socioeconomic factors, and health literacy issues (28).

Our study also indicated that adults aged 65 or older had lower odds of perceiving their health as poorer, with this protective effect being more pronounced in the Hispanic cohort. Recent data on this association among older US adults is limited. However, Axon et al studied the perceived health status of people 50 years or older with self-reported pain that interfered with normal work. Using MEPS data, they found that people aged 65 years or older were more likely than those aged 50 to 64 to perceive their health as good (32). Supporting this finding, an older study from 1983, which conducted telephone interviews with 660 adults in Illinois, found that adults aged 60 or older were more likely than younger adults to have a positive perception of their health (33).

We also observed that women consistently had lower odds than men of perceiving their health as poorer, regardless of their race and ethnicity. This finding supports findings from a study focused on patients diagnosed with heart failure, highlighting the robustness of this association in clinical contexts (34). In a qualitative study involving 32 patients (50% women) from a single outpatient heart failure clinic, women reported better health perceptions than men (34). The better perception of health among women might be due to better psychosocial adjustment of women to their illness, potentially because they ascribe more positive meanings to their condition than men with similar health conditions (34).

These findings underscore the critical need for health care providers to actively address the heightened risk of poor perceived health status among racial and ethnic minority groups with CVD, especially those with physical and cognitive impairments. Health care providers should integrate regular assessments and targeted interventions into care plans, using a team-based model where multidisciplinary teams work together to offer comprehensive, patient-centered care. At the health care system level, prioritizing culturally sensitive care, enhancing health care provider education on health disparities, and incorporating social determinants of health into clinical decision-making are essential steps. Empowering patients through education and fostering strong communication between patients and care teams can further enhance engagement and self-management. By focusing on these areas, health care providers and systems can reduce health disparities and improve outcomes for racial and ethnic minority populations with CVD.

### Strengths and limitations

Although the MEPS is widely recognized as one of the most comprehensive and nationally representative data sources available, it has limitations. Notably, MEPS does not capture information on institutionalized people or people residing in nursing homes, which can introduce a potential source of bias in the data. Additionally, adjustments were necessary in the coding process of MEPS due to limitations in the available digit format for recording health conditions. These modifications may have introduced inaccuracies in estimating the comorbidity scale. Moreover, since MEPS data rely on survey responses from household respondents, there is a risk of recall bias. Furthermore, our study used Andersen’s Behavioral Model of Health Services Use, which emphasizes health care use rather than individual health beliefs or quality of care. Although models like the Health Belief Model or the Theory of Planned Behavior could better capture these aspects, the MEPS dataset lacks the necessary data for their application. Consequently, our findings may not fully reflect the influence of personal health beliefs on health-related quality of life. Similarly, due to MEPS constraints, cultural and contextual variations could not be incorporated into the model. This limitation was a tradeoff for the comprehensive and representative nature of MEPS data, which allowed for a robust analysis despite the absence of these factors. Future research could benefit from integrating these dimensions to provide a more nuanced understanding of disparities.

### Conclusions

This study revealed significant racial and ethnic disparities in perceived health status among adults with CVD and notable differences in how predictive factors affect each racial and ethnic group. To address these disparities, targeted interventions are needed to improve health care access and socioeconomic conditions, specifically tailored to the unique needs and experiences of racial and ethnic groups. These efforts are crucial for promoting equity in health outcomes.

## Appendix Supplemental Tables

**Appendix Table 1:** *International Classification of Diseases* Codes Used for the Identification of Survey Participants With Cardiovascular Disease, Medical Expenditure Panel Survey, 2014–2021

Disease	ICD-9-CM	ICD-10-CM
Ischemic heart disease	410-414	I20-I25
Cardiomyopathy	425	I42, I43
Cardiac arrest	427	I46
Peripheral artery disease	443	I73
Heart failure	428	I50
Arrhythmia	427	I47, I48, I49
Valve problems	397, 424	I07, I34, I35, I37
Stroke	430-434, 436	I60-I69
Ill-defined complications of heart disease	429	I51
Ill-defined cerebrovascular diseases	437	I67
Late effects of cerebrovascular disease	438	I69

Abbreviations: ICD-9-CM, *International Classification of Diseases, Ninth Revision, Clinical Modification*; ICD-10-CM, *International Classification of Diseases, Tenth Revision, Clinical Modification.*

**Appendix Table 2:** *International Classification of Diseases* Codes Used to Calculate a CVD Severity Score Among Survey Participants With Cardiovascular Disease, Medical Expenditure Panel Survey, 2014–2021^a^

Disease	ICD-9-CM	ICD-10-CM
Hypertension	401	I10, I11, I15, I16
Hyperlipidemia	272	E78
Diabetes	250	E10-E14
Proteinuria/albuminuria	791	R80
End-stage renal disease	585	N18
Peripheral vascular disease and atherosclerosis	414, 443	170, I73
Stable angina	413	I20
Cardiac arrest	427^b^	I46
Atrial fibrillation and supraventricular tachycardia	427	I47, I48, I49
Myocardial infarction/acute coronary syndrome	410, 411	I20-I25
Heart valve disease	397, 424^b^	I07, I08, I34, I35, I37
Endocarditis	424	I33
Myocarditis	422	I40, I51
Cardiomyopathy	425	I42, I43
Pericardial disease	423	I30, I31
Ventricular tachycardia/fibrillation^b^	427	I47, I49
TIA/stroke	430-436	G45, I60-I69
Pacemaker or defibrillator use^c^	996	Z95
Congestive heart failure	428	I50

Abbreviations: CVD, cardiovascular disease; ICD-9-CM, *International Classification of Diseases, Ninth Revision, Clinical Modification*; ICD-10-CM, *International Classification of Diseases, Tenth Revision, Clinical Modification.*

^a^ Diseases selected were based on previous literature (15).

^b^ Removed from the score to avoid duplications that could potentially result from the limitations of ICD codes in the Medical Expenditure Panel Survey, 2014–2021.

^c^ Removed from the score due to limited information in the Medical Expenditure Panel Survey, 2014–2021.
